# Prostaglandin E_2_ Production and T Cell Function in Mouse Adenovirus Type 1 Infection following Allogeneic Bone Marrow Transplantation

**DOI:** 10.1371/journal.pone.0139235

**Published:** 2015-09-25

**Authors:** Mary K. McCarthy, Megan C. Procario, Carol A. Wilke, Bethany B. Moore, Jason B. Weinberg

**Affiliations:** 1 Department of Microbiology and Immunology, University of Michigan, Ann Arbor, Michigan, United States of America; 2 Department of Pediatrics and Communicable Diseases, Division of Infectious Diseases, University of Michigan, Ann Arbor, Michigan, United States of America; 3 Department of Internal Medicine, Division of Pulmonary and Critical Care Medicine, University of Michigan, Ann Arbor, Michigan, United States of America; University Paris Sud, FRANCE

## Abstract

Adenovirus infections are important complications of bone marrow transplantation (BMT). We demonstrate delayed clearance of mouse adenovirus type 1 (MAV-1) from lungs of mice following allogeneic BMT. Virus-induced prostaglandin E_2_ (PGE_2_) production was greater in BMT mice than in untransplanted controls, but BMT using PGE_2_-deficient donors or recipients failed to improve viral clearance, and treatment of untransplanted mice with the PGE_2_ analog misoprostol did not affect virus clearance. Lymphocyte recruitment to the lungs was not significantly affected by BMT. Intracellular cytokine staining of lung lymphocytes demonstrated impaired production of INF-γ and granzyme B by cells from BMT mice, and production of IFN-γ, IL-2, IL-4, and IL-17 following ex vivo stimulation was impaired in lymphocytes obtained from lungs of BMT mice. Viral clearance was not delayed in untransplanted INF-γ-deficient mice, suggesting that delayed viral clearance in BMT mice was not a direct consequence of impaired IFN-γ production. However, lung viral loads were higher in untransplanted CD8-deficient mice than in controls, suggesting that delayed MAV-1 clearance in BMT mice is due to defective CD8 T cell function. We did not detect significant induction of IFN-β expression in lungs of BMT mice or untransplanted controls, and viral clearance was not delayed in untransplanted type I IFN-unresponsive mice. We conclude that PGE_2_ overproduction in BMT mice is not directly responsible for delayed viral clearance. PGE_2_-independent effects on CD8 T cell function likely contribute to the inability of BMT mice to clear MAV-1 from the lungs.

## Introduction

Viral infection is an important complication in both allogeneic and autologous bone marrow transplantation (BMT) [1–3]. Human adenoviruses (HAdVs) cause considerable morbidity and mortality in BMT patients [4–6]. Depending on the assay used, HAdVs have been detected in up to 29% of BMT patients during weekly surveillance screening [7,8]. Disease rates as high as 6.5% have been reported, with mortality rates of greater than 50% in BMT patients with HAdV disease in some studies [8,9]. Pediatric patients are at a higher risk for HAdV disease [6,10], likely due to higher infection rates in this population and a relative lack of HAdV species cross-reactive T and B cell responses compared to adults, in whom some HAdV-specific immunity has been established [11]. Severe graft-versus-host disease (GVHD) [12], T-cell-depleted grafts, and leukopenia [13] are additional risk factors for HAdV infection following BMT.

Profound defects in both innate and adaptive immune function are found following BMT [reviewed in [14]]. Both syngeneic and allogeneic BMT mice display defective control of viral replication in a mouse model of respiratory virus infection using murine gammaherpesvirus-68 (γHV-68) [14]. Levels of prostaglandin E_2_ (PGE_2_), a lipid mediator of immune function, are elevated in BMT patients [15,16] and in mouse models of BMT [17–19]. PGE_2_ can be immunosuppressive *in vitro*, inhibiting production of the Th1 cytokines IFN-γ and IL-12 [20,21]. Alveolar macrophage and neutrophil phagocytosis and bacterial killing are also inhibited by PGE_2_ [22,23]. PGE_2_ contributes to the suppression of lymphocyte function observed in human BMT patients [15]. Exaggerated PGE_2_ production and increased susceptibility to *Pseudomonas aeruginosa* and *Staphylococcus aureus* infection occur in syngeneic BMT mice [17–19]. Such mice are unable to effectively control bacterial infections in the lung, and this is directly linked to the immunosuppressive effects of PGE_2_ on macrophage and neutrophil function.

*In vivo* studies of HAdV pathogenesis are limited by the strict species-specificities of the adenoviruses. Inoculation of mice with HAdV does not result in a fully permissive infection [24,25] and therefore does not allow for a complete assessment of host inflammatory responses to adenovirus infection. In contrast, infection of mice with mouse adenovirus type 1 (MAV-1, also known as MAdV-1) serves as an excellent animal model system for studying adenovirus pathogenesis. MAV-1 virions have a morphology that resembles that of HAdV virions [26], and its genomic organization and the majority of its gene products are similar to HAdV counterparts [27–33]. We have worked extensively with MAV-1 as a model to study the pathogenesis of adenovirus respiratory infection [34–41]. MAV-1 targets the respiratory epithelium and replicates in the lungs following intranasal (i.n.) inoculation [35]. Acute MAV-1 respiratory infection causes pulmonary inflammation characterized by a patchy interstitial pneumonitis with scattered areas of hypercellularity around medium and large airways [35]. Interferon (IFN)-γ, interleukin (IL)-17 and other mediators such as prostaglandin E_2_ are upregulated, and effector memory CD4 and CD8 T cells accumulate in the lungs of infected mice [35,37,40,41]. Neonatal mice are more susceptible than adult mice to MAV-1 respiratory infection, with higher lung viral loads, delayed virus clearance from the lungs, and immune responses that are blunted and delayed compared to those in adults [37].

Because HAdV infections are an important complication following allogeneic BMT, we examined pulmonary immunity to adenovirus infection following BMT using MAV-1. After i.n. MAV-1 infection, virus clearance from the lungs was significantly delayed in mice that received allogeneic BMT compared to untransplanted control mice. Our data suggest that T cell function is impaired in BMT mice. Delayed virus clearance was not related to exaggerated PGE_2_ production, because allogeneic BMT using PGE_2_-deficient mice as donors or recipients failed to correct the defect in viral clearance.

## Materials and Methods

### Mice

All animal studies were approved by the University of Michigan Committee on Use and Care of Animals. BALB/c, C57BL/6, CD8α^-/-^ [B6.129S2-*Cd8a* ^*tm1Mak*^/J], IFN-γ^-/-^ [B6.129S7-*Ifng* ^*tm1Ts*^/J], and IFNAR^-/-^ [B6.129S2-*Ifnar1* ^*tm1Agt*^/Mmjax] mice (all knockouts backcrossed onto a C57BL/6 background) were obtained from the Jackson Laboratory (Bar Harbor, ME). Mice heterozygous for microsomal prostaglandin E synthase-1 (mPGES-1) on a DBA1lac/J background (mPGES-1^-/-^ mice) [42] were originally obtained from Pfizer, Inc. (Groton, CT) and then backcrossed onto a C57BL/6 background. Homozygous mPGES-1^-/-^ mice and homozygous wt mPGES-1^+/+^ mice derived from the same heterozygous mPGES-1^+/-^ parents were bred at the University of Michigan. Adult males were used in all experiments. All mice were maintained under specific pathogen-free conditions.

### Bone Marrow Transplantation

BMT was performed as previously described [14,17]. Recipient C57BL/6 mice received 1350 rad of total body irradiation using a ^137^Cs irradiator, delivered in two doses 3 hours apart. In experiments in which C57BL/6 mice were used as donors in the allogeneic transplant, recipient BALB/c mice received 1000 rad of total body irradiation delivered in two doses 3 hours apart. Bone marrow cells (5 x 10^6^) harvested from donor mice were injected into the tail vein of irradiated recipient mice. Mice were given acidified water (pH 3.3) for the first 3 weeks after BMT. Total hematopoietic cell numbers are fully reconstituted in the lung and spleen at 5 weeks post-BMT [14,43]. All infections were carried out 5–6 weeks following BMT. To confirm reconstitution of the lung and spleen in allogeneic BMT mice with donor-derived cells, alveolar macrophages (AM) and splenocytes were harvested from mice at 5 weeks post-BMT. The percentage of cells that were of donor or recipient origin was determined by flow cytometry using antibodies specific for H-2D^b^ (C57BL/6) and H-2D^d^ (BALB/c).

### Virus and Infections

MAV-1 was grown and passaged in NIH 3T6 fibroblasts, and titers of viral stocks were determined by plaque assay on 3T6 cells as previously described [44]. Adult mice were anesthetized with ketamine and xylazine and infected i.n. with 10^5^ plaque forming units of MAV-1 in 40 μl of sterile phosphate-buffered saline (PBS). Control mice were mock infected i.n. with conditioned media at an equivalent dilution in sterile PBS. Mice were euthanized by pentobarbital overdose at the indicated time points. Lungs were harvested, snap frozen in dry ice, and stored at -80°C until processed further.

### Misoprostol treatment

Mice were injected intraperitoneally (i.p.) with 20 μg misoprostol once daily starting on the day of infection, a dosing regimen adapted from [45,46]. Control mice were injected i.p. with an equivalent volume of vehicle (DMSO).

### Isolation of DNA and RNA

DNA was extracted from the middle lobe of the right lung using the DNeasy® Tissue Kit (Qiagen Inc.). Total RNA was extracted from lungs using TRIzol® (Invitrogen) as previously described [39].

### Analysis of Viral Loads

MAV-1 viral loads were measured in organs using quantitative real-time polymerase chain reaction (qPCR) as previously described [37,39]. Primers and probe used to detect a 59-bp region of the MAV-1 E1A gene are detailed in Table 1. Five μl of extracted DNA were added to reactions containing TaqMan II Universal PCR Mix with UNG (Applied Biosystems), forward and reverse primers (each at 200 nM final concentration), and probe (40 nM final concentration) in a 25 μl reaction volume. Analysis on an ABI Prism 7300 machine (Applied Biosystems) consisted of 40 cycles of 15 s at 90°C and 60 s at 60°C. Standard curves generated using known amounts of plasmid containing the MAV-1 EIA gene were used to convert cycle threshold values for experimental samples to copy numbers of EIA DNA. Results were standardized to the nanogram (ng) amount of input DNA. Each sample was assayed in triplicate. The limit of detection of this assay is typically between 10^1^ and 10^2^ copies of MAV-1 genome per 100 ng input DNA.

**Table 1:** Primers and probes used for real-time PCR analysis.

Target	Oligonucleotide	Sequence (5′ to 3′)
MAV-1 E1A	Forward primer	GCACTCCATGGCAGGATTCT
	Reverse primer	GGTCGAAGCAGACGGTTCTTC
	Probe	TACTGCCACTTCTGC
IFN-β	Forward primer	AGCTCCAAGAAAGGACGAACAT
	Reverse primer	GCCCTGTAGGTGAGGTTGATCT
GAPDH	Forward primer	TGCACCACCAACTGCTTAG
	Reverse primer	GGATGCAGGGATGATGTTC

### Analysis of Host Gene Expression

Cytokine gene expression was quantified using reverse transcriptase (RT)-qPCR. First, 2.5 μg of RNA were reverse transcribed using MMLV reverse transcriptase (Invitrogen) in 20 μl reactions according to the manufacturer’s instructions. Water was added to the cDNA product to bring the total volume to 50 μl. Primers used to detect IFN-γ, granzyme B (GzmB), and IFN-β (PrimerBankID 7305123a1 [47]) are described in Table 1. For these measurements, 5 μl of cDNA were added to reactions containing Power SYBR Green PCR Mix (Applied Biosystems) and forward and reverse primers (each at 200 nM final concentration) in a 25 μl reaction volume. When SYBR green was used to quantify cytokine gene expression, separate reactions were prepared with primers for mouse GAPDH (Table 1, used at 200 nM each). In all cases, RT-qPCR analysis consisted of 40 cycles of 15 s at 90°C and 60 s at 60°C. Quantification of target gene mRNA was normalized to GAPDH and expressed in arbitrary units as 2^-ΔCt^, where Ct is the threshold cycle and ΔCt = Ct(target)–Ct(GAPDH).

### Analysis of PGE_2_ Concentration in Bronchoalveolar Lavage Fluid

Mice were euthanized via pentobarbital overdose at the indicated time points. Lungs were lavaged three times with the same aliquot of 1 mL sterile PBS containing protease inhibitor (complete, Mini, EDTA-free tablets; Roche Applied Science). Cells in bronchoalveolar lavage fluid (BALF) were pelleted by centrifugation and supernatant was stored at -80°C until assayed. PGE_2_ concentration in BALF was determined using a PGE_2_ ELISA Kit (Enzo Life Sciences) according to the manufacturer’s protocol.

### Isolation of Cells from Lungs

In some experiments, left lungs were excised and cut into small pieces before digestion for 30 min at 37°C in a 1 mg/ml solution of collagenase A (Sigma). The digested tissue was then pushed through a syringe attached to a 1.5-in 22-gauge needle and pelleted at 3,000 rpm (402 x g) for 5 min. After lysis of red blood cells in 1X lysing buffer (BD PharMingen) for 3 min, tissue debris was removed by a brief spin (~5 to 10 s) at 1,000 rpm (45 x g). The remaining cells were pelleted at 1,200 rpm (64 x g) for 6 min prior to staining.

### Intracellular Cytokine Staining

Cells isolated from lungs were plated at 10^6^ cells/ml and stimulated with 50 ng/ml PMA and 1.5 μM ionomycin (Calbiochem) for 5 h at 37°C. Monensin (Sigma) was added at 3 μM for the last 3 hours of culture. Cells were preincubated with anti-FcγR mAb 2.4G2 to block nonspecific binding before they were stained with the following PE-Cy7-, APC-H7, and V450-conjugated antibodies: CD4 (RM4-5), CD8 (53–6.7), and TCR-β (H57-597) (BD Biosciences). Cells were then fixed in 4% paraformaldehyde for 10 min at room temperature, and permeabilized with 0.2% saponin (Sigma). Finally, cells were stained with FITC- and AF-647-labeled IFN-γ (XMG1.2) and GzmB (GB11) antibodies (BD Biosciences) and analyzed by flow cytometry. Events were acquired on a FACSCanto (BD) flow cytometer, and data were analyzed with FlowJo software (Tree Star). Cells were classified as CD4^+^ T cells (TCRβ^+^CD4^+^) and CD8^+^ T cells (TCRβ^+^CD8^+^).

### Lymphocyte Stimulation

Lymphocytes were seeded at a concentration of 3 x 10^5^ cells/well in 96-well plates coated with anti-CD3 antibody (BioLegend, 5 μg/ml) and incubated for 24 h. Supernatants were then collected for ELISA. Cytokine protein concentrations in supernatant were determined by ELISA (Duoset Kits, R&D Systems) according to the manufacturer's protocol.

### Histology

Lungs were harvested from a subset of mice and fixed in 10% formalin. Prior to fixation, lungs were gently inflated with PBS via the trachea to maintain lung architecture. After fixation, organs were embedded in paraffin and 5 μm sections were obtained for histopathology. Sections were stained with hematoxylin and eosin to evaluate cellular infiltrates. All sectioning and staining was performed by the University of Michigan Comprehensive Cancer Center Research Histology and Immunoperoxidase Laboratory. Slides were viewed through a DM750 microscope (Leica Microsystems). Digital images were obtained with an ICC50 HD digital imaging system (Leica Microsystems) using Leica Acquisition Suite software (Leica Microsystems). Final images were assembled using Adobe Illustrator (Adobe Systems). To quantify cellular inflammation in the lungs, slides were examined in a blinded fashion to determine a pathology index as previously described [34,37].

### Statistics

Analysis of data for statistical significance was conducted using Prism 6 for Macintosh (GraphPad Software, Incorporated). Log transformed values for viral load data were used for statistical comparisons. Differences between groups at multiple time points were analyzed using two-way analysis of variance (ANOVA) followed by Bonferroni's multiple comparison tests. Differences between multiple groups at a single time point were analyzed using one-way ANOVA followed by Bonferroni's or Tukey’s multiple comparison tests. Comparisons between two groups at a single time point were made using the Mann-Whitney rank sum test. Comparisons made between samples from the same mouse treated in two different ways were made using Wilcoxon matched pairs signed rank test. *P* values less than 0.05 were considered statistically significant.

## Results

### Delayed Viral Clearance from Lungs after Allogeneic BMT

**Fig 1:**
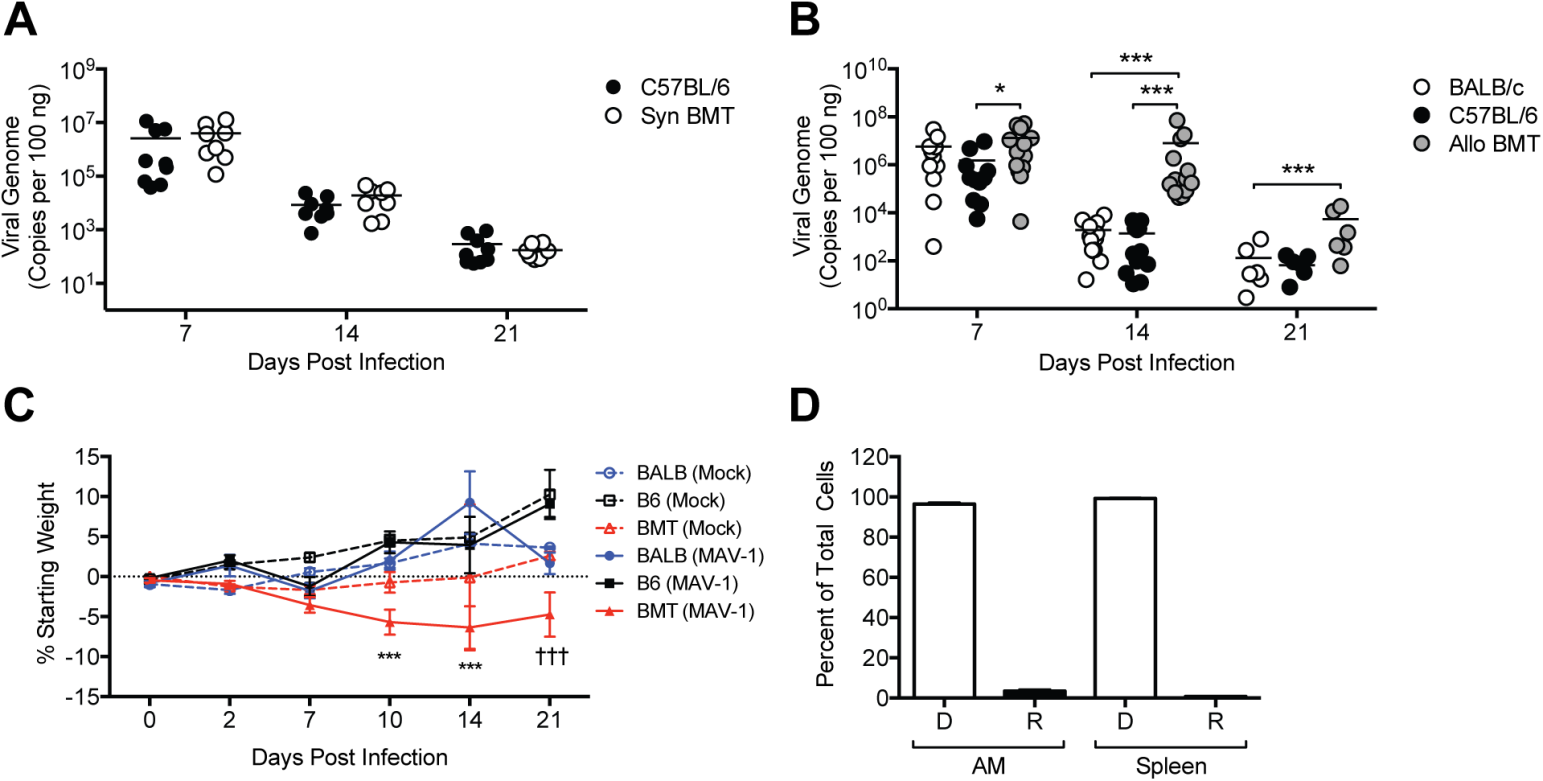
Delayed viral clearance from lungs of mice after allogeneic BMT. A) Syngeneic BMT mice (C57BL/6 donor and recipient) and untransplanted BALB/c and C57BL/6 controls were infected i.n. with MAV-1 at 5 weeks post BMT. DNA was extracted from lungs harvested at the indicated time points. qPCR was used to quantify MAV-1 genome copies in lung DNA. DNA viral loads are expressed as copies of MAV-1 genome per 100 ng of input DNA. B) Allogeneic BMT mice (BALB/c donor, C57BL/6 recipient) and untransplanted BALB/c and C57BL/6 controls were infected i.n. with MAV-1 and lung viral loads were quantified as above. In A and B, individual circles represent values for individual mice (n = 6 to 14 per group per time point) and horizontal bars represent means for each group. Statistical comparisons were made using two-way ANOVA followed by Bonferroni’s multiple comparison tests. ***P<0.001, *P<0.05. C) Weight change was assessed in mock infected and infected allogeneic BMT mice and untransplanted controls (n = 3 to 14 per group, except n = 2 for mock infected B6 mice at 21 dpi). Weight gain or loss is expressed as the percentage of starting weight. ***P<0.001 comparing infected BMT mice to infected C57BL/6 and BALB/c controls; †††*P*<0.001 comparing infected BMT mice to infected C57BL/6 controls. D) In a separate allogeneic BMT experiment (BALB/c donor, C57BL/6 recipient), alveolar macrophages (AM) and splenocytes were harvested from uninfected mice at 5 weeks post-BMT. The percentage of cells that were of donor (D) or recipient (R) origin was determined by flow cytometry using antibodies specific for H-2D^b^ (C57BL/6) and H-2D^d^ (BALB/c). Combined data from n = 3 mice per group are presented as means ± S.E.M.

To determine whether mice are more susceptible to MAV-1 infection following BMT, we first infected untransplanted C57BL/6 mice and syngeneic BMT mice (C57BL/6 donor and recipient) with MAV-1 at 5 weeks post-BMT, when numbers of hematopoietic cells are fully reconstituted [43]. We assessed viral loads in the lung at times corresponding to the peak of viral replication (7 dpi) and clearance of virus from the lungs (14 and 21 dpi). Viral loads were similar in syngeneic BMT mice and untransplanted controls at all times (Fig 1A). To determine whether mice are more susceptible following allogeneic BMT, we infected untransplanted control (BALB/c and C57BL/6) and allogeneic BMT (BALB/c donor, C57BL/6 recipient) mice with MAV-1 at 5 weeks post-BMT. Peak lung viral loads at 7 dpi were significantly higher in allogeneic BMT mice than in C57BL/6 controls at 7 dpi, but this difference was very small (Fig 1B). Viral loads were substantially lower in control mice at 14 and 21 dpi, as virus was cleared from the lungs. However, lung viral loads in allogeneic BMT mice did not substantially decrease from 7 to 14 dpi, and they were significantly greater in allogeneic BMT mice than in control mice at 14 dpi. Lung viral loads in allogeneic BMT mice had decreased by 21 dpi, but they were still significantly greater than those in control mice.

Mock-infected untransplanted control mice, and mock infected BMT mice did not lose weight over the 21-day time course (Fig 1C), suggesting that GVHD was not present in the allogeneic BMT mice. MAV-1 infection did not induce weight loss in untransplanted control mice. In contrast, MAV-1 infection of BMT mice caused significant weight loss that was sustained over the 21-day time course. Reconstitution of the lung and spleen in allogeneic BMT mice with donor-derived cells was confirmed in a representative experiment by staining isolated alveolar macrophages and splenocytes with antibodies specific for H-2D^b^ (C57BL/6) and H-2D^d^ (BALB/c) (Fig 1D). These data indicate that peak viral replication was equivalent in control and allogeneic BMT mice, but viral clearance from the lungs was delayed and infection-induced weight loss was exacerbated in allogeneic BMT mice. Because delayed virus clearance was not present in syngeneic BMT mice, subsequent studies were performed in allogeneic BMT mice.

### MAV-1-Induced Pulmonary Inflammation is Delayed in Allogeneic BMT

**Fig 2:**
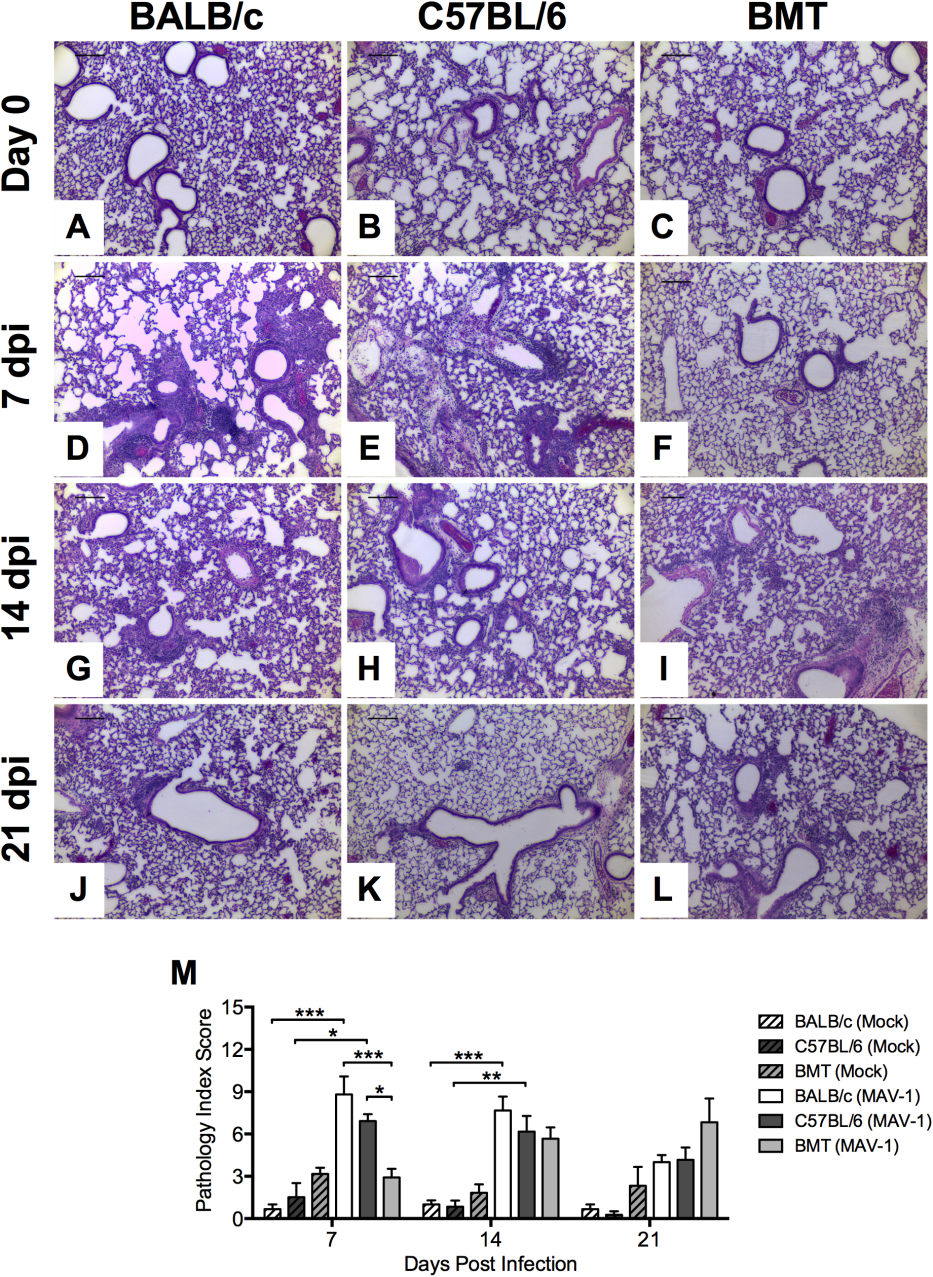
Virus-induced pulmonary inflammation in BMT mice. Allogeneic BMT mice (BALB/c donor, C57BL/6 recipient) and untransplanted BALB/c and C57BL/6 mice were infected i.n. with MAV-1 or mock infected at 5 weeks post BMT. Lungs were harvested and hematoxylin-and-eosin-stained sections were prepared from paraffin-embedded specimens. Representative images are shown from mice before infection (A-C) and from infected mice at the indicated time points (D-L). Scale bars, 100 μm. M) Pathology index scores were generated to quantify cellular inflammation in the lungs of mock-infected and infected mice. Data from 2 to 3 mock-infected mice and 3 to 6 infected mice per group are presented as means and standard errors of the means at each time point. Statistical comparisons were made using two-way ANOVA followed by Bonferroni’s multiple comparison tests. *P<0.05, **P<0.01, and ***P<0.001.

MAV-1 respiratory infection causes pulmonary inflammation characterized by scattered areas of hypercellularity around medium and large airways along with patchy interstitial pneumonitis comprised of a predominantly mononuclear infiltrate and thickened alveolar walls [35,37]. To determine whether delayed virus clearance and enhanced weight loss was associated with altered MAV-1-induced pulmonary inflammation in BMT mice, we evaluated the histological appearance of lungs from mock infected and infected BMT mice and untransplanted controls. Minimal inflammation was present in untransplanted BALB/c and C57BL/6 controls or BMT mice prior to infection (Fig 2A–2C) or in corresponding mock-infected groups at any time post infection (data not shown). Substantial peribronchial and interstitial cellular inflammation was present in BALB/c and C57BL/6 mice at 7 dpi (Fig 2D and 2E), but minimal inflammation was present in BMT mice at 7 dpi (Fig 2F). At 14 dpi, the degree of pulmonary inflammation was similar in lungs of BALB/c, C57BL/6, and BMT mice (Fig 2G–2I). Cellular inflammation had decreased but was still present at 21 dpi in BALB/c, C57BL/6, and BMT mice (Fig 2J–2L). We used a pathology index scoring system to quantify pulmonary inflammation at each time point (Fig 2M). Pathology scores were significantly less in infected BMT mice than in infected BALB/c or C57BL/6 mice at 7 dpi. There were no statistically significant differences in pathology scores in infected BALB/c, C57BL/6, or BMT mice at 14 or 21 dpi.

### PGE_2_ is Overproduced in Allogeneic BMT Infected with MAV-1

**Fig 3:**
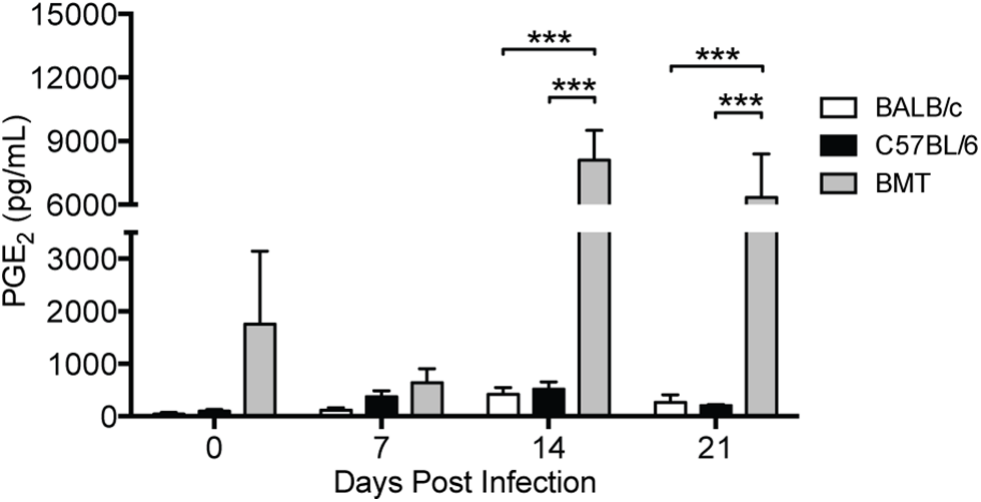
PGE_2_ is overproduced in BMT mice. BMT mice (BALB/c donor, C57BL/6 recipient) and untransplanted BALB/c and C57BL/6 controls were infected i.n. with MAV-1 or mock infected with conditioned media. ELISA was used to quantify PGE_2_ concentrations in BALF at the indicated time points. Combined data from n = 3–4 mice per group are presented as means ± S.E.M. Statistical comparisons were made using two-way ANOVA followed by Bonferroni’s multiple comparison tests. ***P<0.001.

We previously demonstrated exaggerated PGE_2_ production and susceptibility to bacterial infections in syngeneic BMT mice [17,18]. This increased susceptibility is linked to exaggerated PGE_2_ production in BMT mice and to the immunosuppressive effects of PGE_2_ on macrophages and neutrophils. To determine whether allogeneic BMT has a similar effect on PGE_2_ production and MAV-1 pathogenesis, we measured PGE_2_ concentrations in BALF before and after infection of allogeneic BMT mice. Prior to infection (5 weeks post-BMT), BALF PGE_2_ concentrations were somewhat higher in BMT mice than in untransplanted BALB/c and C57BL/6 controls, although this difference was not statistically significant (Fig 3). After infection, PGE_2_ concentrations increased modestly in both untransplanted control groups. In contrast, after infection of BMT mice, BALF PGE_2_ concentrations increased dramatically, and PGE_2_ production was significantly greater in BMT mice than either control group at 14 and 21 dpi.

### PGE_2_ Overproduction Does Not Contribute to Delayed Viral Clearance in BMT Mice

**Fig 4:**
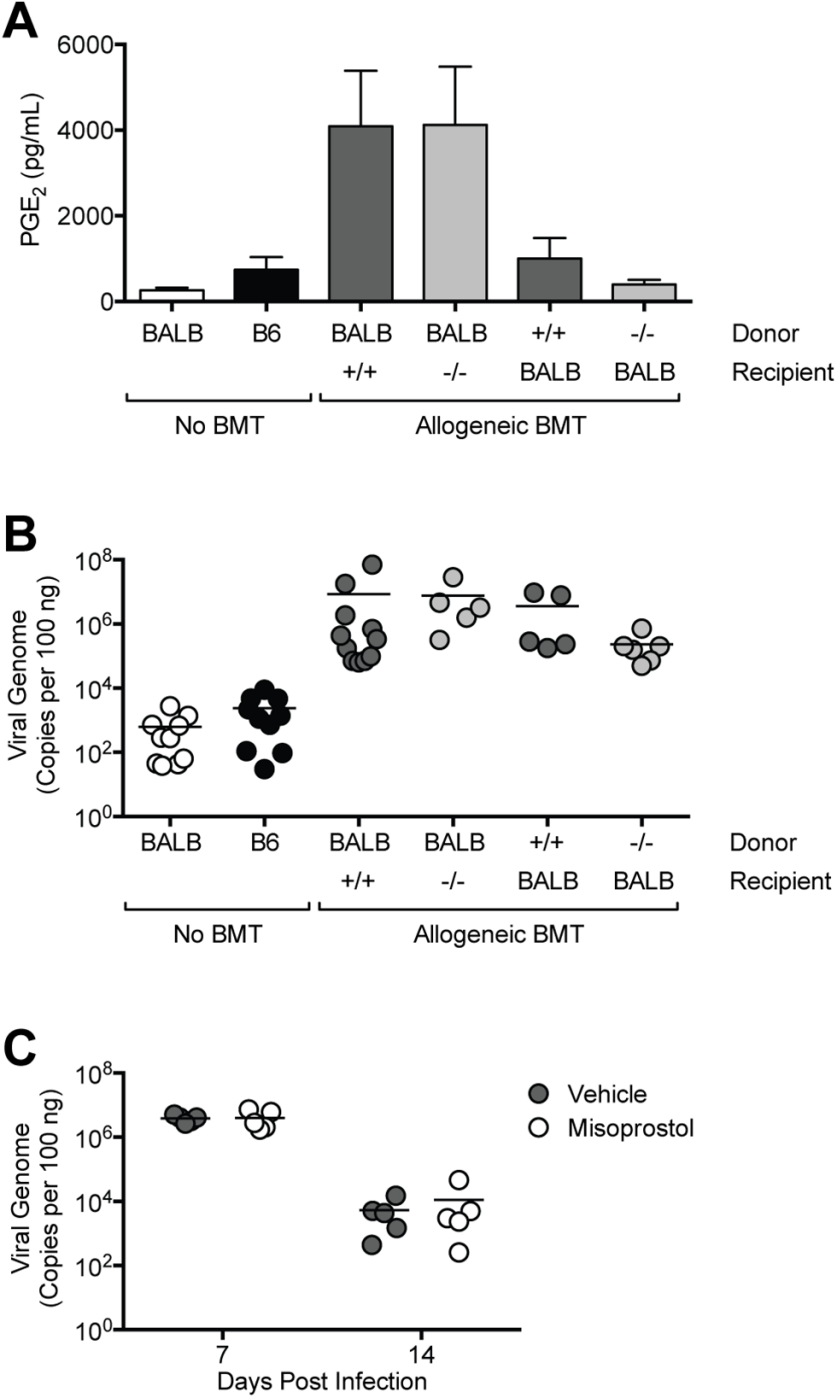
Restoration of normal PGE_2_ levels fails to correct delayed viral clearance in BMT mice. Allogeneic BMT was performed using mPGES-1^+/+^ (+/+) or mPGES-1^-/-^ (-/-) mice on a C57BL/6 background with BALB/c mice in the indicated combinations. BMT mice and untransplanted BALB/c (BALB) and C57BL/6 (B6) controls were infected i.n. with MAV-1 or mock infected with conditioned media. A) ELISA was used to quantify PGE_2_ concentrations in BALF at the indicated time points. Combined data from n = 5–15 mice per group are presented as means ± S.E.M. B) DNA was extracted from lungs harvested at 14 dpi. qPCR was used to quantify MAV-1 genome copies in lung DNA. DNA viral loads are expressed as copies of MAV-1 genome per 100 ng of input DNA. Individual circles represent values for individual mice and horizontal bars represent means for each group. C) C57BL/6 mice were infected i.n. with MAV-1 and treated once daily with 20 μg misoprostol or vehicle (DMSO). DNA was extracted from lungs harvested at the indicated time points. qPCR was used to quantify MAV-1 genome copies in lung DNA. DNA viral loads are expressed as copies of MAV-1 genome per 100 ng of input DNA. Individual circles represent values for individual mice and horizontal bars represent means for each group.

To determine whether PGE_2_ overproduction directly contributes to delayed viral clearance in BMT mice, we performed allogeneic BMT using mPGES-1^-/-^ mice as donors or recipients. mPGES-1 is the terminal synthase responsible for the majority of the conversion of the intermediate PGH_2_ to PGE_2_, so that mPGES-1-deficient mice on a C57BL/6 background (indicated as mPGES-1^-/-^/B6) are almost completely PGE_2_-deficient [48,49]. We infected mice with MAV-1 at 5 weeks post-BMT and measured BALF PGE_2_ at 14 dpi. Virus-induced PGE_2_ was greater in allogeneic BMT mice (BALB/c donor, C57BL/6 recipient), than in untransplanted BALB/c and C57BL/6 control mice (Fig 4A), consistent with our previous observation (Fig 2). When BALB/c bone marrow was transferred into mPGES-1^-/-^/B6 recipients, virus-induced PGE_2_ concentrations were equivalent to those measured in C57BL/6 mice that received BALB/c bone marrow. This suggests that hematopoietic cells from BALB/c donors (which are not PGE_2_-deficient) were the major source of PGE_2_ in infected BMT mice.

To create a situation in which the immune cell compartment was deficient in PGE_2_, we used C57BL/6 or mPGES-1^-/-^/B6 mice as donors and BALB/c mice as recipients. Compared to BMT using BALB/c donors and C57BL/6 recipients, we observed significantly less virus-induced PGE_2_ production at 14 dpi in BMT using C57BL/6 mice as donors and BALB/c mice as recipients (Fig 4A). When mPGES-1^-/-^/B6 bone marrow was transferred into BALB/c mice, virus-induced PGE_2_ production was equivalent to that of either untransplanted control group. The differences in PGE_2_ production observed in BMT combinations using C57BL/6 mice and BALB/c mice as recipients may relate to differing amounts of irradiation received at the time of transplant (1350 rad for C57BL/6 versus 1000 rad for BALB/c) given the higher sensitivity of BALB/c mice to radiation [50]. Despite varying levels of PGE_2_ production, all combinations of allogeneic BMT mice using PGE_2_-deficient mice as donors or recipients had significantly higher lung viral loads at 14 dpi compared to either untransplanted control group (Fig 4B). In addition, we observed no significant differences in virus-induced pulmonary inflammation in all combinations of allogeneic BMT using PGE_2_-deficient mice (data not shown).

To examine the effects of excess PGE_2_ on MAV-1 infection in the absence of other potential effects caused by BMT, we treated untransplanted C57BL/6 mice with misoprostol, a PGE_2_ analog, beginning on the day of infection. Misoprostol treatment did not affect peak viral replication at 7 dpi or viral clearance from the lungs at 14 dpi (Fig 4C). Thus, although PGE_2_ was produced in excess in allogeneic BMT mice following MAV-1 infection, our data collectively indicate that PGE_2_ overproduction did not directly contribute to delayed viral clearance in allogeneic BMT mice.

### T Cell Function is Impaired During MAV-1 Infection of Allogeneic BMT Mice

**Fig 5:**
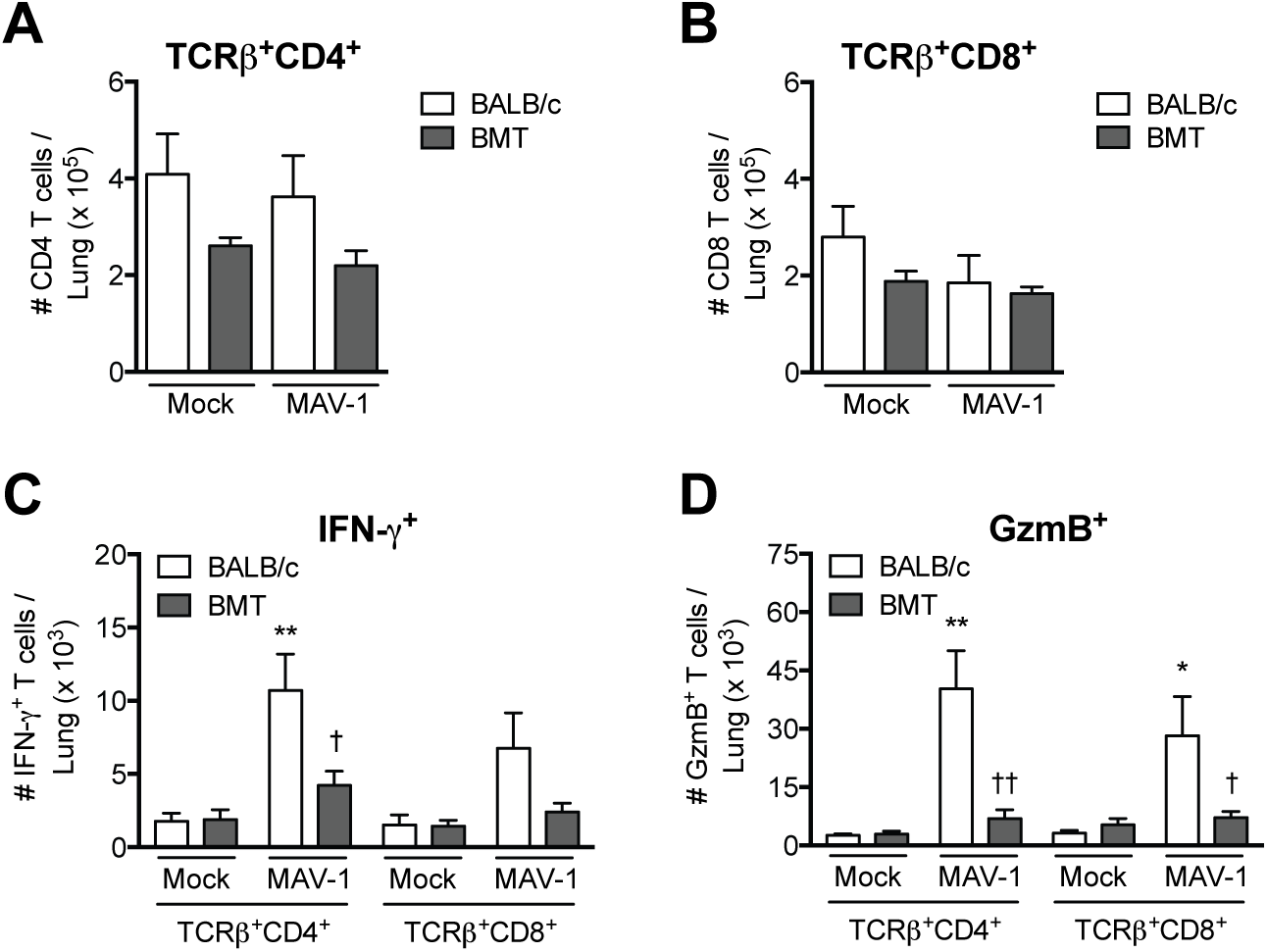
T cells in lungs of BMT mice. BMT mice (BALB/c donor, C57BL/6 recipient) and untransplanted BALB/c and C57BL/6 controls were infected i.n. with MAV-1 or mock infected with conditioned media. Lung leukocytes isolated at 7 dpi were stimulated with PMA/ionomycin and stained to quantify the number of A) TCRβ^+^CD4^+^ T cells, B) TCRβ^+^CD8^+^ T cells, C) IFN-γ^+^ T cells, and D) GzmB^+^ T cells per lung. Combined data from n = 3–4 mice per group are presented as means ± S.E.M. Statistical comparisons were made using one-way ANOVA followed by Bonferroni’s multiple comparison tests. **P<0.01 and *P<0.05 comparing mock to MAV-1. ††*P*<0.01 and †*P*<0.05 comparing BALB/c to BMT mice.

Because delayed virus clearance in BMT mice was not a consequence of PGE_2_ overproduction, we considered other aspects of immune function that could affect MAV-1 clearance during BMT. Impaired CD8 T cell immunity contributes to increased severity of disease caused by Sendai virus in allogeneic BMT mice [51]. We therefore hypothesized that the inability of allogeneic BMT mice to efficiently clear virus from the lungs could be due to impaired T cell recruitment or function during MAV-1 infection. To assess overall CD4 and CD8 T cell recruitment and function, we isolated lung lymphocytes from untransplanted BALB/c and allogeneic BMT mice (in which hematopoietic cells were derived from the BALB/c donor strain) at 7 dpi and used flow cytometry to characterize T cell subsets. There were fewer TCRβ^+^CD4^+^ T cells in the lungs of BMT mice compared to BALB/c mice both before and after infection, although these differences were not statistically significant (Fig 5A). BALB/c and BMT mice had equivalent numbers of TCRβ^+^CD8^+^ T cells in the lungs after infection (Fig 5B). We performed intracellular cytokine staining to evaluate T cell cytokine production at 7 dpi. In untransplanted BALB/c mice, there were more CD4 and CD8 T cells producing IFN-γ (Fig 5C) or GzmB (Fig 5D) after MAV-1 infection. However, numbers of IFN-γ^+^ and GzmB^+^ CD4 and CD8 T cells were significantly less in infected BMT mice. Because there were no significant differences between the numbers of CD8 and CD4 T cells in lungs of BMT mice compared to controls, but the numbers of IFN-γ-producing and GzmB-producing cells were substantially less in BMT mice, our data suggest that T cell function, but not recruitment, was impaired in allogeneic BMT mice infected by MAV-1.

### CD8 T Cells Contribute to MAV-1 Clearance in an IFN-γ-Independent Manner

**Fig 6:**
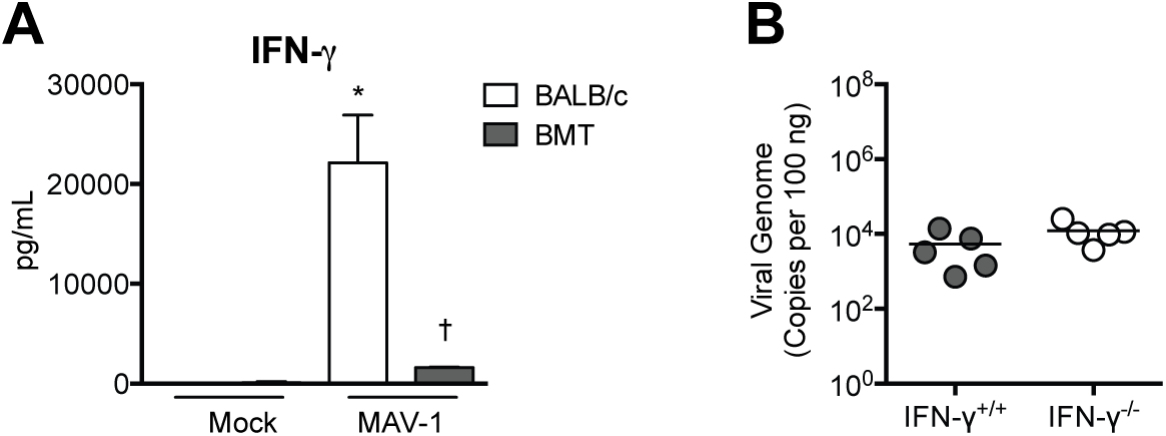
Impaired IFN-γ production in BMT mice. A) BMT mice (BALB/c donor, C57BL/6 recipient) and untransplanted BALB/c controls were infected i.n. with MAV-1 or mock infected with conditioned media, and lung leukocytes were isolated at 7 dpi. Lung leukocytes were stimulated overnight with anti-CD3 antibody and ELISA was used to measure IFN-γ concentrations in supernatants. Combined data from n = 3–8 mice per group are presented as means ± S.E.M. Statistical comparisons were made using one-way ANOVA followed by Tukey’s multiple comparison tests. **P<0.01 and *P<0.05 comparing mock to MAV-1. ††*P*<0.01 and †*P*<0.05 comparing BALB/c to BMT mice. B) A) IFN-γ^+/+^ and IFN-γ^-/-^ mice were infected i.n. with MAV-1. DNA was extracted from lungs harvested at 14 dpi. qPCR was used to quantify MAV-1 genome copies in lung DNA. DNA viral loads are expressed as copies of MAV-1 genome per 100 ng of input DNA. Individual circles represent values for individual mice and horizontal bars represent means for each group. Statistical comparisons were made using the Mann-Whitney test.

To further address T cell function in allogeneic BMT mice, we isolated lung lymphocytes from BMT mice and untransplanted BALB/c controls at 7 dpi, restimulated the cells overnight with anti-CD3 antibody, and measured IFN-γ concentrations in supernatant following restimulation. Cells isolated from infected, untransplanted BALB/c mice produced significantly more IFN-γ upon restimulation than cells isolated from mock infected BALB/c mice (Fig 6A). To determine the extent to which deficient IFN-γ responses in BMT mice contributed to delayed clearance of MAV-1, we infected untransplanted IFN-γ^+/+^ and IFN-γ^-/-^ mice and measured lung viral loads at 14 dpi. Viral loads were equivalent in IFN-γ^+/+^ and IFN-γ^-/-^ mice (Fig 6B), suggesting that decreased IFN-γ production by lymphocytes in BMT mice did not play a significant role in delayed viral clearance. Similarly, we have shown that IFN-γ deficiency on a BALB/c background leads to only a modest (<1 log) increase in MAV-1 viral loads at 14 dpi [37].

**Fig 7:**
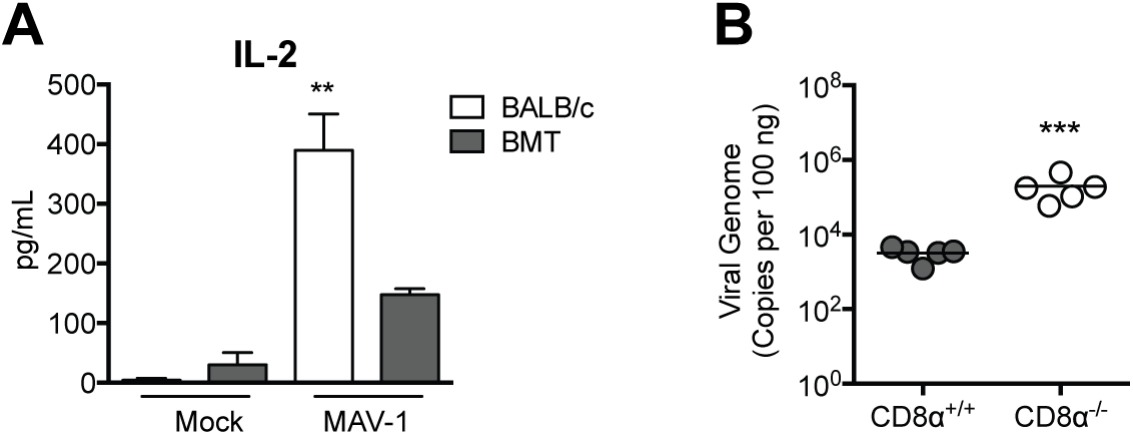
Delayed viral clearance in the absence of CD8 T cells. A) BMT mice (BALB/c donor, C57BL/6 recipient) and untransplanted BALB/c controls were infected i.n. with MAV-1 or mock infected with conditioned media, and lung leukocytes were isolated at 7 dpi. Lung leukocytes were stimulated overnight with anti-CD3 antibody and ELISA was used to measure IL-2 concentrations in supernatants. Combined data from n = 3–8 mice per group are presented as means ± S.E.M. Statistical comparisons were made using one-way ANOVA followed by Tukey’s multiple comparison tests. **P<0.01 and *P<0.05 comparing mock to MAV-1. B) CD8α^+/+^ and CD8α^-/-^ mice were infected i.n. with MAV-1. DNA was extracted from lungs harvested at 14 dpi. qPCR was used to quantify MAV-1 genome copies in lung DNA. DNA viral loads are expressed as copies of MAV-1 genome per 100 ng of input DNA. Individual circles represent values for individual mice and horizontal bars represent means for each group. Statistical comparisons were made using the Mann-Whitney test. ***P<0.001.

We also observed that production of IL-2 (Fig 7A) along with IL-4 and IL-17 (S1 Fig) by lung lymphocytes isolated from BMT mice was impaired compared to production by lung lymphocytes isolated from untransplanted BALB/c controls. We have previously demonstrated that MAV-1 lung viral loads are not affected by IL-17 deficiency [40], so effects of BMT on IL-17 production are unlikely to have contributed to delayed viral clearance in BMT mice. However, because IL-2 makes critical contributions to CD8 T cell proliferation, differentiation, and effector function [52], this further suggested that CD8 T cell function was impaired in BMT mice. To determine whether aspects of CD8 T cell function other than IFN-γ production were required for viral clearance, we infected CD8α^+/+^ and CD8α^-/-^ mice with MAV-1 and measured lung viral loads at 14 dpi. Viral loads were significantly greater in CD8α^-/-^ mice compared to CD8α^+/+^ mice (Fig 7B). These results indicate that CD8 T cells contribute to clearance of virus from the lung during MAV-1 infection, although not via production of IFN-γ. Taken together, these data suggest that CD8 T cell dysfunction contributed to delayed virus clearance in BMT mice.

### Type I IFN Responses Do Not Contribute to Delayed Virus Clearance in BMT Mice

**Fig 8:**
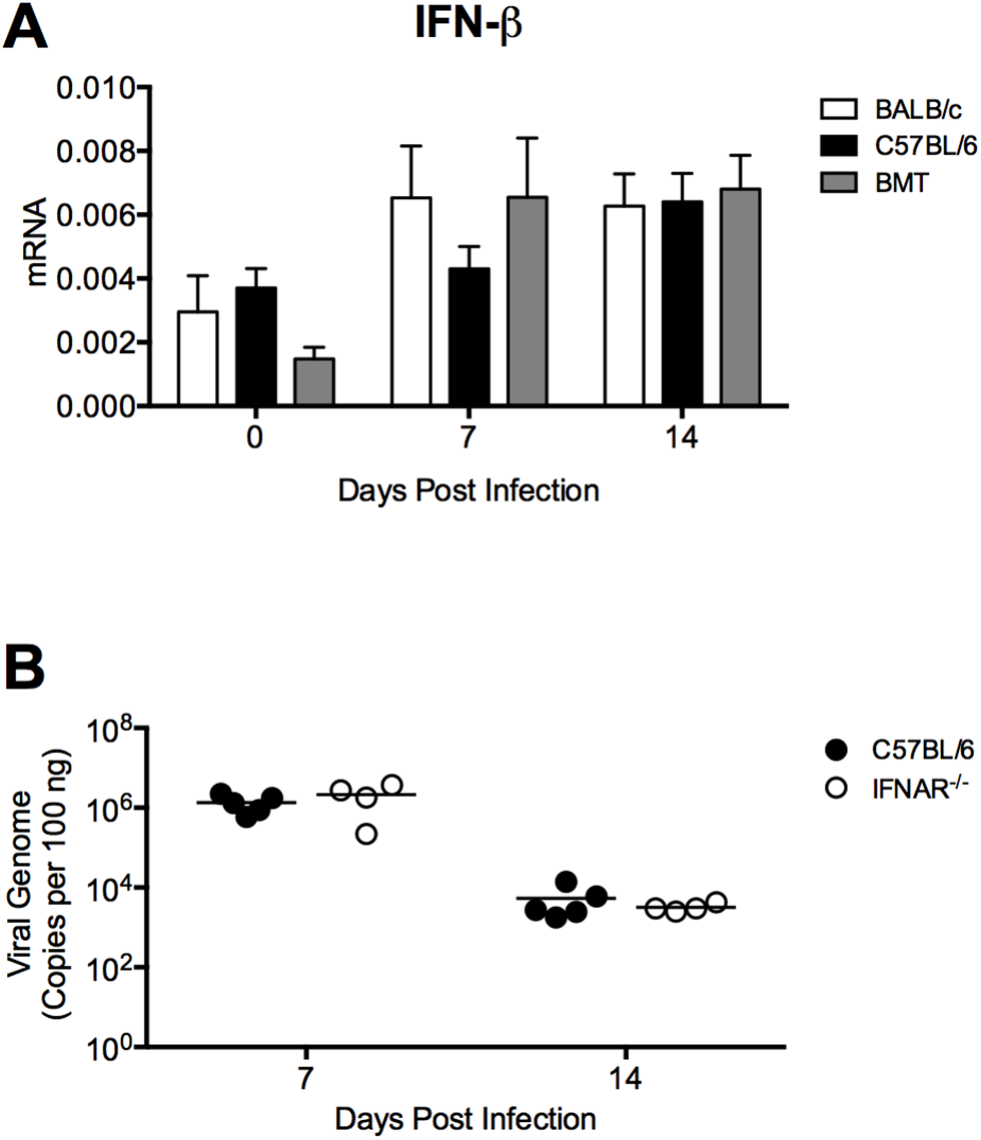
Type I IFN production in BMT mice. A) BMT mice (BALB/c donor, C57BL/6 recipient) and untransplanted BALB/c and C57BL/6 controls were infected i.n. with MAV-1, and lungs were harvested at the indicated time points. RT-qPCR was used to quantify IFN-β mRNA levels. Combined data from n = 7–8 mice per group (n = 3 per group at day 0) are presented as means ± S.E.M. Statistical comparisons were made using two-way ANOVA followed by Bonferroni’s multiple comparison tests. B) Untransplanted C57BL/6 and IFNAR^-/-^ mice were infected i.n. with MAV-1. DNA was extracted from lungs harvested at the indicated time points. qPCR was used to quantify MAV-1 genome copies in lung DNA. DNA viral loads are expressed as copies of MAV-1 genome per 100 ng of input DNA. Individual circles represent values for individual mice and horizontal bars represent means for each group. Statistical comparisons were made using two-way ANOVA followed by Bonferroni’s multiple comparison tests.

To determine whether allogeneic BMT modulated type I IFN responses in a way that could affect MAV-1 pathogenesis, we used RT-qPCR to quantify mRNA levels of IFN-β in the lungs of BMT mice and untransplanted controls (Fig 8A). IFN-β mRNA levels were slightly lower in BMT mice than in untransplanted controls prior to infection, but this difference was not statistically significant. There were no statistically significant differences in IFN-β mRNA levels between BMT mice and untransplanted controls at any time point (Fig 8A). In addition, we infected untransplanted mice deficient in type I IFN signaling (IFNAR^-/-^ mice) and B6 controls and measured lung viral loads at 7 and 14 dpi (Fig 8B). Viral loads were equivalent in B6 and IFNAR^-/-^ mice at each time point. Thus, our data indicate that type I IFN activity is not essential for control of MAV-1 replication or for clearance of virus from the lungs.

## Discussion

HAdV infection is a potentially devastating complication in hematopoietic stem cell transplant (HSCT) patients, and there are no antiviral or immunomodulatory therapies that have consistently shown a benefit in HSCT patients with HAdV disease. We used MAV-1 to study the effects of BMT on host susceptibility to adenovirus infection. Mice that underwent allogeneic BMT displayed significant defects in clearance of MAV-1 from the lungs. This altered susceptibility in allogeneic BMT mice was independent of pharmacologic immunosuppression that is typically used in transplant recipients. It occurred at a time post-BMT at which significant GVHD, a risk factor for HAdV infection in some reports [12] was not present in the allogeneic BMT mice ([14] and data not shown). However, syngeneic BMT had no effect on clearance of MAV-1 from the lungs. Thus, allogeneic BMT itself was associated with intrinsic changes in host immune function that impaired control of MAV-1 infection in the lung.

Concentrations of the immunomodulatory lipid mediator PGE_2_ were dramatically higher in BALF from allogeneic BMT mice than in untransplanted controls after MAV-1 infection. However, allogeneic BMT using PGE_2_-deficient mice as donors or recipients failed to correct the defect in viral clearance, and treatment of untransplanted mice with the PGE_2_ analog misoprostol was not associated with increased lung viral loads. T cell recruitment to the lungs was not significantly affected by BMT, similar to our previous findings in syngeneic BMT mice [14]. BMT mice had significantly fewer IFN-γ-producing and GzmB-producing CD4 and CD8 T cells in the lungs compared to untransplanted controls, and cytokine production by restimulated T cells was substantially lower in T cells isolated from BMT mice compared to controls. Lung viral loads in untransplanted CD8-deficient mice were higher than in wt mice, suggesting that a defect in CD8 T cell function contributed to delayed MAV-1 clearance in BMT mice.

We previously demonstrated that acute MAV-1 infection increases PGE_2_ production in the lungs of untransplanted C57BL/6 mice, but PGE_2_ deficiency (in mPGES-1^-/-^ mice) has little effect on the amount of viral replication in the lungs, clearance of virus from the lungs, or virus-induced lung inflammation [41]. In this study, we addressed the question of whether exaggerated virus-induced PGE_2_ production in BMT, a scenario that is distinct from induction of PGE_2_ production in an immunocompetent host, would affect MAV-1 pathogenesis. We detected dramatically increased PGE_2_ in the airways following MAV-1 infection of allogeneic BMT mice, similar to findings from other studies using mouse models of bacterial infection [17–19] or γHV-68 infection [53] following syngeneic BMT. PGE_2_ suppresses the production of Th1 cytokines [20,21], an effect that is observed when local PGE_2_ concentrations are greater than 1 nM [54]. Others have demonstrated PGE_2_ inhibition of T cell proliferation in mixed lymphocyte reactions (MLR) [55]. PGE_2_ inhibits T cell recruitment and delays the induction of virus-specific T cell responses in the lungs of influenza virus-infected mice [56]. We hypothesized that overproduction of PGE_2_ in allogeneic BMT mice was responsible for suppression of T cell responses that normally clear virus from the lung. However, BMT using PGE_2_-deficient mice as donors or recipients did not correct the defect in viral clearance. The amount of PGE_2_ detected in those experiments varied based on donor and recipient background strain, most likely due to differences in the amount of irradiation used for BALB/c versus C57BL/6 recipients at the time of transplant. However, virus clearance was delayed in all transplanted mice despite the absence of increased PGE_2_. In addition, untransplanted mice treated with the PGE_2_ analog misoprostol cleared virus as efficiently as controls. Thus, while PGE_2_ is overproduced after allogeneic BMT and suppresses T cell responses in other settings, our data suggest that PGE_2_ overproduction does not contribute to delayed clearance of MAV-1 following allogeneic BMT.

While PGE_2_ overproduction was apparently not the cause of T cell dysfunction, our data indicate that aberrant T cell function in allogeneic BMT mice is a potential explanation for delayed virus clearance. This is similar to findings in a recent study indicating that impaired clearance of Sendai virus in allogeneic BMT is due to T cell dysfunction [51]. In our study, CD8α^-/-^ mice had higher viral loads than CD8α^+/+^ mice at 14 dpi, suggesting that CD8α^-/-^ mice had a defect in clearance of virus from the lungs similar to that observed in allogeneic BMT mice. Although allogeneic BMT mice had significantly fewer IFN-γ-producing CD8 T cells and less IFN-γ production following lymphocyte restimulation, IFN-γ^-/-^ mice cleared virus from the lungs as efficiently as IFN-γ^+/+^ controls. Similarly, we have previously shown that IFN-γ makes only minor contributions to control of MAV-1 replication in another mouse strain background, BALB/c [37]. It is therefore unlikely that delayed virus clearance in allogeneic BMT mice was a direct consequence of BMT-associated changes in T cell IFN-γ production. Likewise, changes in type I IFN production are not likely to explain delayed virus clearance. Rather, delayed MAV-1 clearance from the lungs of BMT mice may be due to alterations in other aspects of cytotoxic T cell function (e.g. GzmB, perforin or Fas/FasL signaling), since MAV-1 clearance was similarly impaired in CD8α^-/-^ mice but not IFN-γ^-/-^ mice. It is possible that CD8 T cell dysfunction in allogeneic BMT mice infected with MAV-1 was due to enhanced suppression via interactions between programmed death 1 and PD-1 ligand proteins, as is the case with Sendai virus infection in allogeneic BMT [51]. Future studies will systematically address which CD8 effector functions are most critical for clearance of MAV-1 as well as the specific mechanisms underlying T cell dysfunction post-BMT.

The difference between lung viral loads at 14 dpi in CD8α^-/-^ and CD8α^+/+^ mice (Fig 7B, approximately 2 log) was not as great as the difference between BMT mice and controls (Fig 1B, approximately 4 log). This suggests that impairment of other aspects of host immune function is likely to contribute to delayed clearance of MAV-1. The requirement for IFN-γ production to control viral clearance is pathogen-specific. While CD4 T cell-derived IFN-γ is important for clearance of lytic γHV-68 infection in BMT mice [14], our results suggest that IFN-γ is not required for clearance of MAV-1 from the lungs. Although contributions of CD4 T cells to clearance of MAV-1 from the lungs have yet to be fully defined, we previously demonstrated that lung viral loads are slightly higher in MHC class II-deficient mice at 7 dpi [41]. It is therefore possible that CD4 T cell dysfunction also contributes to delayed MAV-1 clearance in allogeneic BMT mice, but this is unlikely to be directly related to IFN-γ production by CD4 T cells. Although it is possible that antigen presenting cell dysfunction may have contributed to some of the observed effects, we have previously demonstrated that antigen presenting cell function is intact in syngeneic BMT mice [14].

We conclude that allogeneic BMT is associated with PGE_2_ overproduction, T cell dysfunction, and impaired clearance of MAV-1 from the lungs. However, excess PGE_2_ does not directly contribute to the delayed virus clearance. It is possible that increased viral replication at late time points, resulting from delayed virus clearance in BMT mice due to T cell dysfunction, was actually the cause of exaggerated PGE_2_ production instead of its indirect consequence. Our results emphasize the importance of immune dysfunction that is intrinsic to allogeneic BMT, distinct from effects of immunosuppressive medications used in transplant recipients. Our findings are similar to those in transplant recipients infected with HAdV. Immune recovery appears to play a significant role in HAdV infections post-transplantation. A number of studies have documented a positive correlation between lymphocyte count (both absolute lymphocyte count and CD4 count) and clearance of HAdV and survival [13,57,58]. In adult and pediatric HSCT patients, clearance of HAdV from peripheral blood is associated with the emergence of HAdV-specific CD4 and CD8 T cell responses [59]. Clearance of HAdV is also associated with an increase in titers of serotype-specific antibodies [57], indicating that both B and T cell function may be important for control of HAdV post-transplantion.

*Ex vivo* generation and adoptive transfer of virus-specific CD4 or CD8 T cells has been explored as immunotherapy for HSCT patients with disease caused by HAdV and other viruses. This strategy has been successfully used in transplant patients with Epstein Barr virus-induced lymphoproliferative disease [60–62] and with some success in pediatric transplant patients with HAdV viremia [63] or prophylactically to prevent HAdV disease [64]. A number of studies have demonstrated *ex vivo* generation of HAdV-specific cytotoxic CD4 and CD8 T cells, some of which were cross-reactive against multiple HAdV serotypes [65–70]. *Ex vivo* generation of single-culture T cells specific for HAdV and five other viruses has also been used as an approach to treat transplant patients infected with multiple viruses at the same time [71]. These studies show promise for the effective treatment of HAdV infections post-HSCT. The results of our work with MAV-1 in BMT expand our understanding of adenovirus pathogenesis in an immunocompromised host. Our model is well suited for future work to improve preventative and treatment strategies used for transplant recipients with disease caused by HAdV and other pathogens.

## Supporting Information

S1 Dataset Complete data for all experiments described in this manuscript are included in the file “BMT and MAV-1 Manuscript PLOS ONE Complete Data 081115.xlsx.” (XLSX)

S1 Fig Impaired Cytokine production in BMT mice. BMT mice (BALB/c donor, C57BL/6 recipient) and untransplanted BALB/c controls were infected i.n. with MAV-1 or mock infected with conditioned media, and lung leukocytes were isolated at 7 dpi. Lung leukocytes were stimulated overnight with anti-CD3 antibody and ELISA was used to measure concentrations of A) IL-4 and B) IL-17 in supernatants. Combined data from n = 3–8 mice per group are presented as means ± S.E.M. Statistical comparisons were made using one-way ANOVA followed by Tukey’s multiple comparison tests. **P<0.01 comparing mock to MAV-1. ††*P*<0.01 comparing BALB/c to BMT mice.
(TIFF)
