# Supplementary figures and images for: Prostaglandin E2 Production and T Cell Function in Mouse Adenovirus Type 1 Infection following Allogeneic Bone Marrow Transplantation
Source: PLoS One. 2015 Sep 25;10(9):e0139235. doi: 10.1371/journal.pone.0139235 (PMC4583312; doi:10.1371/journal.pone.0139235)

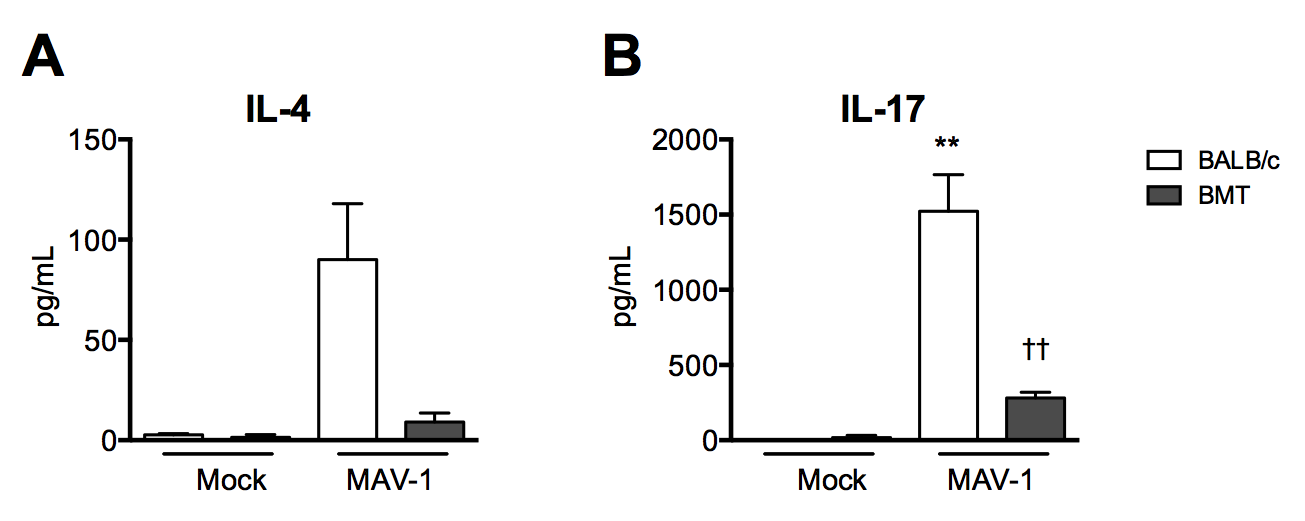

Supplement: S1 Fig — BMT mice (BALB/c donor, C57BL/6 recipient) and untransplanted BALB/c controls were infected i.n. with MAV-1 or mock infected with conditioned media, and lung leukocytes were isolated at 7 dpi. Lung leukocytes were stimulated overnight with anti-CD3 antibody and ELISA was used to measure concentrations of A) IL-4 and B) IL-17 in supernatants. Combined data from n = 3–8 mice per group are presented as means ± S.E.M. Statistical comparisons were made using one-way ANOVA followed by Tukey’s multiple comparison tests. **P<0.01 comparing mock to MAV-1. ††P<0.01 comparing BALB/c to BMT mice. (TIFF) [file pone.0139235.s002.tiff]
